# Mathematical model of blood glucose dynamics by emulating the pathophysiology of glucose metabolism in type 2 diabetes mellitus

**DOI:** 10.1038/s41598-020-69629-0

**Published:** 2020-07-29

**Authors:** Nelida Elizabeth López-Palau, José Manuel Olais-Govea

**Affiliations:** 1 División de Matemáticas Aplicadas, IPICyT, Camino a la Presa San José No. 2055, Lomas Cuarta Sección, 78216 San Luis Potosí, SLP Mexico; 2 Tecnologico de Monterrey, Escuela de Ingeniería y Ciencias, Av. Eugenio Garza Sada 300, 78211 San Luis Potosí, SLP Mexico; 3 Tecnologico de Monterrey, Writing Lab, TecLab, Vicerrectoría de Investigación y Transferencia de Tecnología, 64849 Monterrey, NL Mexico

**Keywords:** Computational biology and bioinformatics, Diseases, Mathematics and computing

## Abstract

Mathematical modelling has established itself as a theoretical tool to understand fundamental aspects of a variety of medical-biological phenomena. The predictive power of mathematical models on some chronic conditions has been helpful in its proper prevention, diagnosis, and treatment. Such is the case of the modelling of glycaemic dynamics in type 2 diabetes mellitus (T2DM), whose physiology-based mathematical models have captured the metabolic abnormalities of this disease. Through a physiology-based pharmacokinetic-pharmacodynamic approach, this work addresses a mathematical model whose structure starts from a model of blood glucose dynamics in healthy humans. This proposal is capable of emulating the pathophysiology of T2DM metabolism, including the effect of gastric emptying and insulin enhancing effect due to incretin hormones. The incorporation of these effects lies in the implemented methodology since the mathematical functions that represent metabolic rates, with a relevant contribution to hyperglycaemia, are adjusting individually to the clinical data of patients with T2DM. Numerically, the resulting model successfully simulates a scheduled graded intravenous glucose test and oral glucose tolerance tests at different doses. The comparison between simulations and clinical data shows an acceptable description of the blood glucose dynamics in T2DM. It opens the possibility of using this model to develop model-based controllers for the regulation of blood glucose in T2DM.

## Introduction

For some decades, mathematical models have been used in biological sciences to understand diverse aspects of *diabetes mellitus* (DM)^1^. For example, DM progression^2,3^, diagnostic test evaluations^4,5^, long-term micro and macrovascular complications^6,7^, and blood glucose dynamics^8–10^, among others, have been modeled. Particularly, mathematical models to emulate blood glucose dynamics in DM have been classified, according to the complexity of their description, in two major groups^11^. The first group considers the whole-body models developed under a pharmacokinetic–pharmacodynamic (PKPD) approach, which is characterized by being structurally simple with a limited physiological interpretation. The second group considers the physiological based PKPD (PB-PKPD) models, which mathematically describe the physiological interactions between different subsystems of the human body. Due to its structural simplicity, most of the models in the literature are PKPD^1^. Although these models are widely used, they do not include most of the processes responsible for glucose homeostasis. Hence, its use to model complex processes in DM, such as the DM pathophysiology, is limited and, it induces a trend toward the development of PB-PKPD models^1^. These models have focused on emulating the metabolic processes involved in glucose homeostasis, and are usually organ-based. Moreover, the PB-PKPD models of blood glucose dynamics in type 1 DM (T1DM) have been useful to synthesize model-based controllers for blood glucose regulation in T1DM^12–16^. However, type 2 DM (T2DM) affects multiple subsystems of the body and, consequently, the mathematical representation of the metabolic abnormalities in T2DM is challenging^17^.

One of the most widely used PB-PKPD models was performed by Sorensen^10^. This organ-based compartmental model emulates the blood glucose dynamics of a healthy human by considering the main glucose metabolic rates as mathematical functions. In this model, each mathematical function was individually fitted to a set of clinical data of healthy people where the metabolic response of the patients was measured for different stimuli. Then, the physiology of the main metabolic rates of a healthy human body was mathematically reproduced. Although Sorensen’s model is quite robust, it has some limitations. For instance, the model does not include blood glucose and insulin dynamics in the pancreas. Instead, a single function representing the pancreatic insulin release rate is connected to the bloodstream. The above does not represent the physiology of the human body. In addition, the model does not consider the effect of gastric emptying. Therefore, the blood glucose dynamics after oral glucose intake, and the potentiating-insulin effect of the incretin hormones cannot be reproduced.

An extension of the Sorensen’s model, which covers its main limitations, was proposed by Alverhag and Martin^9^. Thus, the model included two ordinary differential equations (ODE) to quantify, through mass balance, the time-variation of the blood glucose and insulin in the pancreas. Additionally, the gastric emptying process, and the enhancing effect on insulin due to the incretin hormones were included by considering two new subsystem attached to the model. Furthermore, Alverhag and Martin hypothesize that a model of the blood glucose dynamics in T2DM can be developed by identifying the parameters of the mathematical functions representing the metabolic rates related to the pathophysiology of this condition^9^. Based on the above, Vahidi et al. used a nonlinear optimization approach to identify some parameters of the Sorensen’s model from a single data set of an oral glucose tolerance test (OGTT) in T2DM patients^8^. Even though in this article, the system response acceptably reproduces the OGTT, the set of identified parameters that minimize the error between clinical data and the system may not be unique. Therefore, it cannot be assured that the metabolic functions containing the identified parameters emulate the pathophysiology of the T2DM individually.

Consequently, this article proposes a PB-PKPD model of the blood glucose dynamics in T2DM, where some mathematical functions representing metabolic rates of the body, are individually fit to emulate the pathophysiology of the T2DM. Moreover, the effect of the gastric emptying, and the enhancing effect of insulin due to the incretin hormones are included to reproduce the blood glucose dynamics after oral glucose intake. To achieve this, the mathematical model of the blood glucose dynamics in a healthy human body, proposed in Alvehag and Martin^9^, will be described as a set of 28-dimensional ODE. From the ODE set, the mathematical functions representing the impaired metabolic rates in T2DM were individually fitted to clinical data of T2DM patients by using the least-squares method (LSM). The clinical data were taken from several clinical tests where direct measurements of the tissues or organ response to local changes in solutes concentration were made. The resulting model was numerically simulated to test its ability to reproduce the blood glucose dynamic in T2DM patients for different inputs, and initial conditions. Finally, the error between the simulation, and the clinical data of the T2DM patients is quantified by using a statistical function.

This manuscript is organized as follows: “Methodology" shows the methodology, while the results and discussions are set out in “Results and discussion”. Finally, the article ends with some concluding observations on this work in “Concluding remarks”. Also, Supplementary information have been included to show the nomenclature of all the variables and the numerical values of the parameters contained in the equations used throughout the manuscript.

## Methodology

The mathematical model in Alverhag y Martin is a nonlinear dynamic system consisting of four clustered subsystems^9^. The subsystems are compartmental representations of the human body, where each compartment represents an organ or tissue where an important process of mass exchange is carried out. The compartments are interconnected through the blood flow. Then, by means of a mass balance in the compartments, each of the subsystems quantifies the concentration of one solute (i.e., glucose, insulin, glucagon, or incretins). A detailed explanation of the system and its nomenclature can be found the Supplementary information.

The system is a set of 28 ODEs composed of nonlinear continuous functions. Therefore, it follows that the solution of the system (*x*(*t*)) exists in a domain $\mathbb {D}$ as long as the initial conditions are in $\mathbb {D}$. As a methodological approach in this work, the solution of the system is represented from a state-space theory as the vector:

1 $$\begin{aligned} x= \; &[G_{BV},\; G_{BI},\; G_H,\; G_L,\; G_K, G_{PV},\; G_{PI},\; G_G,\; G_{PN}, \nonumber \\&I_B,\; I_H,\; I_L,\; I_K,\; I_{PV},\; I_{PI},\; I_G,\; I_{PN},\; \Gamma ^N,\; \omega ,\; \omega _G, \nonumber \\&M_{HGP}^I,\; M_{HGU}^I,\; F_2,\; P,\; I,\; Q,\; G_s,\; r_{OGA}] \end{aligned}$$

where $x(t)=(x_1(t), x_2(t), \ldots , x_{28}(t))\in \mathbb {D}\subset \mathbb {R}^{28}$ is semidefined positive, which means that it belongs to the set $\mathbb {R}^{28}_{+}$. Using the state definition in Eq. (1), the system is defined as:

2 $$\begin{aligned} \dot{x}(t)=F(x(t);\pi ,\eta ), \ \ x(t_0)=x_0 \in \mathbb {D} \end{aligned}$$

where the vector field $$F(x(t);\pi , \eta ): \rightarrow \mathbb {R}^{28}$$ determines the time evolution of *x*(*t*) starting at initial condition ($$x_{0}$$) in the initial time ($$t_{0})$$, and $$\pi \in \Pi \subset \mathbb {R}^{46}$$ contains the parameters in the functions representing hemodynamical processes, while $$\eta \in $$H$$\subset \mathbb {R}^{67}$$ contains the parameters in the functions representing the metabolic rates of the system. The parameter values of the system in Eq. (2) can be found in the Supplementary information.

### Model simulation and initialization

The mathematical model in Eq. (2) successfully simulates the blood glucose dynamics of a healthy human body after intravenous glucose infusion and oral glucose intake^9^. For the above, an input to the system is considered containing: (i) a continuous intravenous glucose infusion rate ($r_{IVG}$), which is introduced to the system as an insulin rate in mg$\cdot$(dL$\cdot$min)$^{-1}$, and (ii) an oral glucose intake ($OGC_{0}$), which is introduced to the system in mg and it is connected to the gastric emptying process (see the Supplementary information). The output of the system (*y*) is considered as $x_6=G_{PV}$ and $x_{14}=I_{PV}$, whose meaning concerns to glucose and insulin vascular concentration in peripheral tissues, respectively. The time evolution of *y* is used to compare the model simulation with clinical data where the glucose and insulin concentrations are taken from a blood sample of the patient’s forearm during a test. For all the simulations, the model in Eq. (2) was numerically solved by using a variable step in the function *ode45 (Dormand-Prince)* of MATLAB^18^. The simulation time was defined as the time length of the clinical trial.

For model initialization, the basal condition $x^B$ and $x_0$ were computed from the solute concentrations in the fasting state of the patients. The condition $x^B$ is determined as the mean fasting glucose and insulin concentration from the blood samples collected over several days, this is $x_6^B$ and $x_{14}^B$, respectively. The condition $x_0$ is determined as the fasting glucose and insulin concentrations from a blood sample at time zero of the clinical test; this is $x_6(0)$ and $x_{14}(0)$, respectively. Mathematically, the fasting state has a physiological correspondence with the steady-state of the system ($x^*$) in Eq. (2), this is:

3 $$\begin{aligned} F(x^*;\pi ;\eta ) = 0 \end{aligned}$$

then, since interstitial, arterial, and venous concentrations are the same at the steady-state, the peripheral vascular data for $x^B$ and $x_0$ are computed from the arterial or venous data. The remaining 26 components of $x^B$ and $x_0$ are obtained from the solution of the Eq. (3).

### Metabolic rates of the model

The subsystems described in the Supplementary information are coupled by the functions representing the metabolic rates of the glucose, insulin, glucagon, and incretins. These metabolic rates are mathematically modeled as constant or linear functions of the mass accumulation in the compartments; or multiplicative functions of the metabolic basal rate. Specifically, the metabolic rates in the glucose, and glucagon subsystems are multiplicative functions with the following general form:

4 $$\begin{aligned} r=M^G M^I M^{\Gamma } r^B \end{aligned}$$

where $r^B$ represents the basal value of the metabolic rate *r*, and each *M* is the isolated effect of the normalized concentration of glucose ($M^G$), insulin ($M^I$), and glucagon ($M^{\Gamma }$) of the normalized metabolic rate ($r^N=r/r^B$). The above implies that $M^G=M^I=M^\Gamma =1$ when the glucose, insulin, and glucagon are basal, therefore $r=r^B$. To represent the characteristic sigmoidal non-linearities of biological data correlations, excepting the isolated effects that are states of the system in Eq. (2) (i.e., $M_{HGP}^I$ and $M_{HGU}^I$), all the isolated effects are hyperbolic tangent functions of some normalized component of the state, this is:

5 $$\begin{aligned} M(x_i^N)=\eta _{j_1}+\eta _{j_2}\tanh (\eta _{j_3}(x^N_i+\eta _{j_4})) \end{aligned}$$

where $x^N_i=x_i/x_i^B$ for $i\in {\{1, 2, \ldots 28\}}$, and $\eta _{j_1}, \eta _{j_2}, \ldots ,\eta _{j_4} \in H$ with $j_1, j_2 \ldots j_4 \in \mathbb {N} \le 67$ are dimensionless parameters. A list containing the nominal values of the $\eta$ parameters can be found in the Supplementary information. Using these values, the system in Eq. (2) simulates the blood glucose dynamics after an intravenous glucose infusion or an oral glucose intake in a healthy human body^9^. For the mathematical modelling of the blood glucose dynamics of T2DM, the pathophysiology of T2DM must be emulated by modifying the value of the parameters of the functions representing the metabolic rates responsible of the characteristic hyperglycaemia. The above will be described in “Curve fitting”.

### Curve fitting

For decades, different studies have identified the metabolic problems associated with the progression of T2DM in healthy humans^19,20^. It has been found that these problems are related to the metabolism of fats, and carbohydrates^19,20^. The metabolism of this latter is the object of study in this work.

Mainly, the pathophysiology of the T2DM is characterized by^19^: (i) insulin resistance, defined as an impaired effect of insulin on glucose uptake by peripheral tissues, (ii) excessive hepatic glucose production, due to accelerated gluconeogenesis, and (iii) $\beta$-cell dysfunction, represented by an impaired pancreatic insulin release. Then, the mathematical functions of the system in Eq. (2) modelling the aforementioned metabolic rates are: the effect of insulin in peripheral glucose uptake (i.e., $M_{PGU}^I$), the effect of glucose, insulin, and glucagon on the hepatic glucose production (i.e., $M_{HGP}^{G}$, $M_{HGP}^{I_{\infty }}$ and $M_{HGP}^{\Gamma _0}$, respectively), and the pancreatic insulin release (i.e., $r_{PIR}$). Since a small variation in the parameters of the after-mentioned metabolic rates results in a variation of the solute concentrations in the model, in the following sections, the terminology of the sensitivity analysis from Khalil will be adopted^21^. Therefore, the above metabolic rates will be called *sensitive metabolic rates*.

In what it follows, the sensitive metabolic rates were selected to fit the clinical data of T2DM patients. Explicitly, the fitting of $r_{PIR}$ is supported by several clinical tests where a decrease of the first phase of pancreatic insulin release in patients with T2DM is exhibited^22–24^. The above is consistent with the early proposal to induce a partial impairment on insulin release from the labil compartment, in order to decrease the first phase of insulin release in T2DM patients^25^. Due to the above, the functions representing the first phase of insulin release (*X* and $P_\infty$), and the time-variation of the amount of labile insulin ready to be released, were studied by a sensitivity analysis as in Khalil^21^ to select the parameters that show a major contribution to the sensitivity on solution $x(t;\eta ,\pi _0)$. The selected parameters were identified from the clinical data of T2DM patients. The rest of the parameters remained unaltered.

#### Static and dynamic fitting approach

To solve the parameter fitting problem, two things are required:

1. A set of clinical data in T2DM patients.
2. A mathematical method to fit such data to the function representing the sensitive metabolic rates.

The set of clinical data used for the isolated effects fitting was obtained from selected clinical tests of T2DM patients. The conditions of each one of the selected articles are consistent with those originally considered for mathematical modeling in Ref.^10^. These conditions are compiled in Table 1. In the selected articles, the clinical data was taken from a set $n_p$ of individual with no other significant medical history than T2DM. Nevertheless, for curves fitting, we used the reported mean value of the tissue/organ response to local changes in the solute concentration of the $n_p$ subjects. Originally, to mathematically model the metabolic rate $r_{PIR}$, Grodsky obtained data from a graded glucose step-response with the isolated perfused pancreas in rats^25^. Since it is impossible to obtain this data from humans, the selected parameters of this metabolic rate were identified using clinical data from an input–output approach of the system, in Eq. (2). The data were taken from an OGTT in DeFronzo et al.^26^, where the plasma glucose and insulin response to oral intake were measured in nine T2DM subjects after the consumption of 1 g/kg-body weight of oral glucose.

**Table 1:** Conditions of the clinical test and its interpretation in the mathematical model.

Rate	Conditions
$M_{PGU}^I$	The glucose concentration was maintained in its basal state by a glucose clamp (i.e., $M_ {PGU}^G=1$). The above allows to observe the isolated effect of insulin in the peripheral glucose uptake (i.e., $M_{PGU}^I$) by knowing the normalized value of the peripheral glucose uptake (i.e., $r_{PGU}^N=r_{PGU}/r_{PGU}^B$)
$M_{HGP}^G$	Somatostatin was administered to block the endogenous release of insulin, and glucagon. Exogenous insulin, and glucagon replacements were introduced to the patients to maintain them in their basal state (i.e., $M_{HGP}^\Gamma =1, x_{21}=x_{21}^*$ ). The above allows to observe the isolated effect of glucose on the hepatic glucose production (i.e., $M_{HGP}^{G}$) by knowing the normalized value of hepatic glucose production (i.e., $r_{HGP}^N=r_{HGP}/r_{HGP}^B$)
$M_{HGP}^{I_{\infty }}$	Glucose concentration was maintained at basal state by means of a glucose clamp. The above allows to observe the isolated effect of insulin in the hepatic glucose production after a stabilization time (i.e., $M_{HGP}^{I_{\infty }}$)
$M_{HGP}^{\Gamma _0}$	Somatostatin was administered to block the endogenous release of insulin, and glucagon. Exogenous insulin, and glucose replacements were introduced to the patients to maintain them in their basal state by means of a glucose clamp (i.e., $M_{HGP}^G=1,x_{21}=x_{21}^*$ ). The above allows to observe the isolated effect of glucagon on the hepatic glucose production (i.e., $M_{HGP}^{\Gamma _0}$) by knowing the normalized value of hepatic glucose production (i.e., $r_{HGP}^N=r_{HGP}/r_{HGP}^B$)

The clinical data from the studies that fulfill the criteria in the table were used to fit the isolated effects of the sensitive metabolic rates of the model.

The mathematical method used to fit the functions to clinical data is the least squares (LSM). In general, the LSM lies that the following relation is fulfilled^27^:

6 $$\begin{aligned} \bar{y}=g(z, \theta ) \end{aligned}$$

where *z*, and $\bar{y}$ are vectors containing *n* observations, and $\theta \in \mathbb {R}^{p \times 1}$ is a vector of *p* unknown parameters of the sensitive metabolic rate. To estimate $\theta$ the *n* values of *g* are computed for all *z*. Then, $\hat{\theta }$ is the estimation of the vector of parameters corresponding to $\theta$ that minimizes the residual sum of squares of an objective function $Q(\theta )$ over some feasible the vector of parameters $\theta \ge 0 \subset \Theta$. The isolated effects of the sensitive metabolic rates were fitted to clinical data by a static approach of the LSM. After that, a dynamical approach of the LMS was used to identify the parameters of the $r_{PIR}$ function. In what follows, both approaches will be described.

In the static approach, the unknown parameters from the Eq. (5) are grouped as $\theta =[\eta _{j_1}, \eta _{j_2}, \eta _{j_3}, \eta _{j_4}]^T$. The vector $\hat{\theta }$ is estimated with an iterative process using the following objective function:

7 $$\begin{aligned} Q(\theta )=\sum _{k=1}^n \left( y_k-M\left( z_k, \theta \right) \right) ^2 \end{aligned}$$

where $y_k$ is clinical data of the mean of the normalized metabolic rate in T2DM patients respect its basal value in Ref.^9^, and $z_k$ is the clinical data of the mean of the normalized solute concentration taken from the forearm. The minimization of the objective function in Eq. (7) was numerically solved with the function *lsqcurvefit* of the optimization toolbox of MATLAB^18^. The iterative algorithm used to find $\hat{\theta }$ was ‘trust-region reflective’ proposed in Li^28^. After fitting, ($z_k$,$y_k$) are graphically compared with the fitted isolated effects functions. Then, the values of the parameters in $\theta$ were replaced by the values in $\hat{\theta }$.

In the dynamical approach the selected parameters from $r_{PIR}$ were grouped as $\theta = [\eta _{l_1}, \eta _{l_2}, \eta _{l_3}, \eta _{l_4}, \eta _{l_5}, \eta _{l_6}]^T$ with $l_1, l_2, \ldots l_6 \in \mathbb {N} \le 67$. The vector $\hat{\theta }$ was estimated with an iterative process using the following objective function:

8 $$\begin{aligned} Q(\theta )=\sum _{k=1}^{n} \left( \left( \frac{y_{1k}-f_1(z_k, \theta )}{w_1}\right) ^2 + \left( \frac{y_{2k}-f_2(z_k, \theta )}{w_2} \right) ^2 \right) ^{1/2} \end{aligned}$$

where $y_{1k}$, and $y_{2k}$ are the clinical data obtained from the mean of glucose and insulin concentrations, respectively, taken at the $z_k$ time, the weights $w_1$ and $w_2$ are the mean of the basal glucose, and insulin concentrations, respectively; and $f_1=x_6(z_k,\theta )$, $f_2=x_{14}(z_k,\theta )$ were obtained from the model simulation. The above clinical data was taken from DeFronzo et al.^26^. The LSM problem in Eq. (8) was numerically solved using the function *fmincon* of the optimization toolbox of MATLAB^18^ with the iterative algorithm ‘interior-point’. After the identification of the parameters of $r_{PIR}$, the values in $\theta$ (from the static, and dynamical approach) were replaced by $\hat{\theta }$ in order to emulate the pathophysiology of T2DM. Hereinafter, the resulting model is called *T2DM model*.

### Comparison of the T2DM model with clinical data

The T2DM model was numerically simulated for comparison with a clinical test in T2DM where the blood glucose dynamics is observed after different stimuli. Considering that the route of glucose entry into the body plays an essential role overall glucose homeostasis^26^, the T2DM model was simulated for the following test: (i) a programmed graded intravenous glucose infusion test (PGIGI) to account for the rapid response of the intravenous infusions, and (ii) an OGTT considering a dose of 50 g of glucose (50 g-OGTT), and a dose of 75 g of glucose (75 g-OGTT) to account for blood glucose changes due to the gastric emptying process, and the effects of the incretin.

The clinical data used to compare the DMT2 model with a PGIGI test was obtained from Carperntier et al.^29^. In this test, the glucose was administered intravenously in a total of 7 subjects with DMT2 (i.e., $n_p = 7$). Mathematically, this is that the glucose was supplied through $r_ {IVG}$ while $OGC_0 = 0$. The duration of the test was 270 min distributed as follows: a basal sampling period was considered were $r_{IVG}=0$ from 0 to 30 min, after this, the steps of intravenous glucose infusion were introduced as $r_{IVG}=1, 2, 3, 4, 6$, and 8 mg (dL min)$^{-1}$ for a period of 40 min each one. The conditions for model simulation were $G_{PV}^B=G_{PV}(0)=157.5$ mg dL$^{-1}$, and $I_ {PV}^B=I_{PV}(0)=13.02$ mU $\hbox {L}^{-1}$.

The clinical data used to compare the DMT2 model with an OGTT was obtained from Firth et al.^30^, and Mari et al.^31^. In these test, 50 and 75 g of oral glucose was consumed by a total of 13 and 46 subjects with DMT2, respectively (i.e., $n_p=13$ or $n_p=46$). Mathematically, this is that the glucose was supplied through $OGC_0$ while $r_{IVG}=0$. For the OGTT, the duration of the simulation was 180 min. The conditions for model simulation were $OGC_0=$50,000 mg, $G_{PV}^B=G_{PV}(0)=185$ mg dL$^{-1}$, and $I_{PV}^B=I_{PV}(0)=14$ mU L$^{-1}$, for the 50 g-OGTT. Further, the conditions for model simulation were $OGC_0=$75,000 mg, $G_{PV}^B=G_{PV}(0)=176$ mg dL$^{-1}$, and $I_{PV}^B=I_{PV}(0)=11.2$ mU L $^{-1}$, for the 75 g-OGTT.

The difference between the clinical data, and the model simulation was quantified with the following statistical expression:

9 $$\begin{aligned} \sigma =\root \of {\frac{1}{n-1}S_e} \end{aligned}$$

where $S_e=\sum _{s=1}^n(x_6(t_s)-G(t_s))^2$, and *G* is the glucose concentration taken from the T2DM patients at the time $t_s$. All the clinical tests were different from those used for parameter fitting.

### Declarations

The source of clinical data was obtained from publicly available sources, namely, recognized research journals and properly cited through the manuscript. No person was directly involved in this study as a source of clinical data.

## Results and discussion

**Figure 1:**
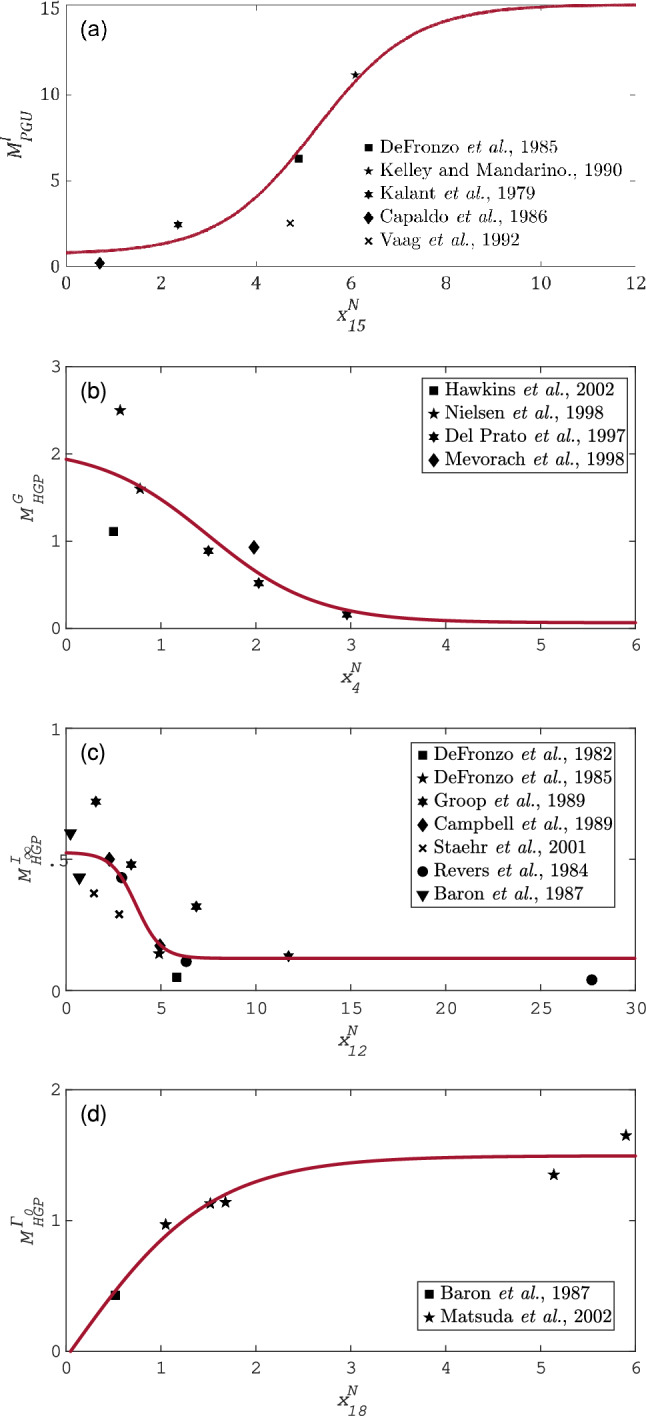
Isolated effects fitting to clinical data. In these plots the solid line represents the isolated effects functions (**a**) $M^I_{PGU}$, (**b**) $M^G_{HGP}$, (**c**) $M^{I_\infty }_{HGP}$, and (**d**) $M^{\Gamma _0}_{HGP}$ fitted to the clinical data from T2DM patients. Each symbol represents the mean measured value of the tissue/organ response to a local change on the solute concentration, from $n_p$ subjects. For these metabolic rates, the fitting approach was static.

The clinical data that fulfill the conditions provided in Table 1 were taken from the references grouped in Table 2. The parameter set $\hat{\theta }$ for each isolated effect of the sensitive metabolic rate can be seen in Table 3. Furthermore, in Fig. 1 it can be found a graphic representation of the curves that fit the isolated effects functions of the sensitive metabolic rates to the clinical data of the Table 1. As can be seen, the curves in Fig. 1 do not necessarily pass through the point $(x_i^N,M^N(x_i))=(1,1)$. This is because the isolated effects of the metabolic rates were normalized with respect to the basal value of the metabolic rates in Alverhag and Martin^9^, which correspond to a mathematical model of the blood glucose dynamics in a healthy human body. The above is justified by the fact that not all isolated effects of glucose, insulin, or glucagon on a metabolic rate are observed altered in T2DM patients. Since the metabolic rates are expressed as multiplier factors of the basal metabolic rate, the isolated effects that have not been observed altered in patients with T2DM will continue to be multiplier factors of the basal metabolic rate ($r^B$) of a healthy human body.

**Table 2:** References of the clinical studies.

Rate	References	$n_p$
$M_{PGU}^I$	DeFronzo et al.^26^	9
	Vaag et al.^32^	12
	Kelly and Mandarino^33^	15
	Capaldo et al.^34^	6
	Kalant et al.^35^	11
$M_{HGP}^G$	Hawkins et al.^36^	10
	Mevorach et al.^37^	9
	Nielsen et al.^47^	9
	Del Prato et al.^48^	9
$M_{HGP}^{I_{\infty }}$	Staehr et al.^49^	10
	Groop et al.^38^	9
	Campbell et al.^39^	14
	Baron et al.^41^	10
	DeFronzo et al.^26^	9
	Revers et al.^40^	10
	DeFronzo et al.^50^	38
$M_{HGP}^{\Gamma _0}$	Matsuda et al.^42^	8
	Baron et al.^41^	10

The table shows the set of references containing the clinical data used to fit the isolated effects of the sensitive metabolic rates. Column $n_p$ indicates the number of patients analyzed in each reported clinical study according to the reference in the central column of the table. The proposed parametric adjustment results from taking the means of each set of $n_p$ patients.

**Table 3:** Vector of fitted parameters from the static approach.

$M(z_k,\hat{\theta })$	$\hat{\theta }$
$M_{PGU}^I(x_{15}^N,\hat{\theta })$	$[7.9869, 7.2537, 0.4852, -5.2518]$
$M_{HGP}^G(x_{4}^N,\hat{\theta })$	$[1.0720, -1.0064, 0.8712, -1.4930]$
$M_{HGP}^{I_{\infty }}(x_{12}^N,\hat{\theta })$	$[0.3240, -0.2020, 0.7625, -3.6977]$
$M_{HGP}^{\Gamma 0}(x_{18}^N,\hat{\theta })$	$[0, 1.495, 0.6773, -0.0469]$

The table shows the parameter values that minimize the residual sum of squares of the objective function for the different isolated effects.

As can be seen in Fig. 1a the curve corresponding to $M_{PGU}^I$ goes close to the point ($x_{15}^N$, $M_{PGU}^I(x_{15}^N))=(1,1)$. The above means that the insulin-stimulated peripheral glucose uptake in a T2DM patient does not differ much from the one in a healthy human when the fasting hyperglycaemia, and basal insulin concentration are maintained in the T2DM patient. This characteristic of the T2DM has been previously reported in several articles^32–35^. In contrast, as can be seen in Fig. 1b, for $x_4^N=1$, the value of $M_{HGP}^G$ is higher than one. Considering basal hyperglycaemia, it means that the hepatic glucose production is higher in T2DM patients compared to that observed in healthy humans. The above has been previously reported by various articles where the effect of glucose on the hepatic glucose production rate was verified for healthy control subjects, and T2DM patients. Hawkins et al.^36^ have associated this increase with accelerated gluconeogenesis since glycolysis is normal for healthy subjects and diabetic subjects. Besides, Mevorach et al.^37^ report that this inefficient suppression is due to a deficient inhibition of glucose-6-phosphatase activity and/or lack of inhibition of glucose-6-phosphate formation.

The characteristic hepatic insulin resistance of the T2DM is evident in Fig. 1b,c. This can be observed in the behavior of the curves for high values of the solute concentration, where the hepatic glucose production can not be suppressed entirely despite significant increment of the normalized glucose and insulin concentration in the liver. The above is consistent with clinical evidence where the blood glucose has an impaired ability to inhibit the hepatic glucose production at basal insulin and glucagon concentrations in T2DM^19,36,37^; and the insulin concentration is ineffective to suppress the hepatic glucose production at basal glucose and glucagon concentrations in T2DM^26,38–40^. Finally, the role of glucagon in hepatic glucose production in T2DM patients can be seen in Fig. 1d. In this graphical representation, the behavior of the function $M_{HGP}^{\Gamma _0}$ is consistent with the clinical data of patients with T2DM^41,42^. Then, it follows that by fitting the isolated effect functions to the clinical data, it is possible to individually emulate the pathophysiology of the T2DM.

After isolated effects fitting, a parameter set containing the parameters of $r_{PIR}$ that shows a greater contribution to the sensitivity on the solution $x(t;\eta ,\pi _0)$ was selected. From the sensitive analysis, the selected set of parameters was $\hat{\theta }= [\eta _{36}, \eta _{39}, \eta _{40}, \eta _{42},$ $\eta _{44}, \eta _{45}]^T$. The values of $\hat{\theta }$ that minimize the objective function in Eq. (8) can be seen in Table 4.

**Table 4:** Vector of identified parameters from the dynamical approach.

$f(z_k,\hat{\theta })$	$\hat{\theta }= [\eta _{36}, \eta _{39}, \eta _{40}, \eta _{42}, \eta _{44}, \eta _{45}]^T$
$x_6(t,\hat{\theta })$	[3.2717, 2.8504, 0.9330, 0.0867, 7.6707, 0.0565]
$$x_{14}(t,\hat{\theta })$$	

The table shows the parameter values that minimize the residual sum of squares of the objective function for the sensitive metabolic rate $r_{PIR}$.

**Figure 2:**
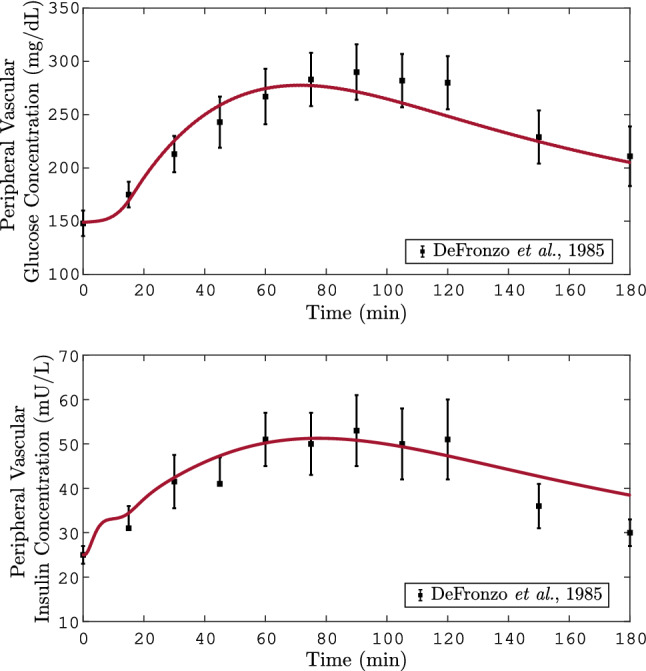
Graphical result of $r_{PIR}$ fitting to the clinical data. In these plots the solid line represents the variation of the (**a**) glucose or (**b**) insulin concentration in the peripheral compartment of the T2DM model. The symbols represent the mean±SEM value of the solute from the $n_p$ subjects. These data were taken from DeFronzo et al. where a 70 g-OGTT was performed^26^. For the simulation it was considered a consumption of 70 g of glucose at time equal to zero. The $r_{PIR}$ parameters are those whom minimized the objective function from the dynamic fitting approach.

Once the nominal values of the parameters are replaced by those in $\hat{\theta }$ of Tables 3, and 4, the system simulates the response to a 70 g-OGTT. The results of the simulation, and its comparison with clinical data from DeFronzo et al.^26^ can be seen in Fig. 2. As can be seen there, is an acceptable approximation of the simulation curve to the clinical data. Moreover, during almost all the simulation time, the model output remains within the bars of the standard error. It should be noted that even when $y_{1k}$, and $y_{2k}$ have different orders of magnitude, the emulation of both blood glucose and insulin was successfully achieved. This is mainly due to the addition of weight functions of weights in the objective function of the Eq. (8).

As noted in “Methodology", since there is no clinical data of the individual response of $r_{PIR}$ measured against different stimuli, the dynamic approach used to fit $r_{PIR}$ is based on nonlinear optimization. As a result, the set of values obtained minimizes the objective function of the Eq. (8), nevertheless, it cannot be assured that the pathophysiology of the pancreatic insulin secretion in T2DM is individually emulated. However, due to the individual fitting of the isolated effects, the number of parameters to be identified by a dynamic approach is minimal. A proposal to avoid the above is to replace the pancreatic insulin subsystem with a model of the pancreas whose pathophysiology could be described by a set of clinical data of patients with DMT2.

**Figure 3:**
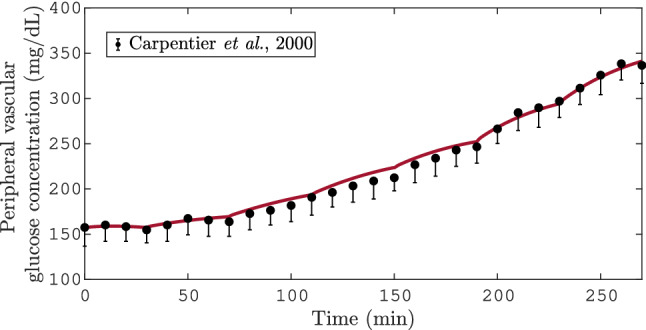
Simulation of a PGIGI test. In this plot the solid line represents the simulation of the blood glucose in the peripheral compartment of the T2DM model. The symbols represent the mean±SEM value of solute from the $n_p$ subjects. These data were taken from Carpentier et al. where a PGIGI test was performed^29^.

After the metabolic rates fitting the resulting model (i.e., T2DM model) was simulated and compared with clinical data. In Fig. 3, it can be seen the T2DM model response for the PGIGI test, and the clinical data from Carpentier et al.^29^. As can be seen, the simulation of the T2DM model is not significantly different from the reported clinical data. Moreover, the absolute of the maximum difference between simulation, and the clinical data is 9.4 mg dL$^{-1}$. This is consistent with the obtained statistical value $\sigma =5.37$ mg dL$^{-1}$ for this test. It follows that the T2DM model can reproduce the step response of the blood glucose due to an intravenous glucose infusion input.

**Figure 4:**
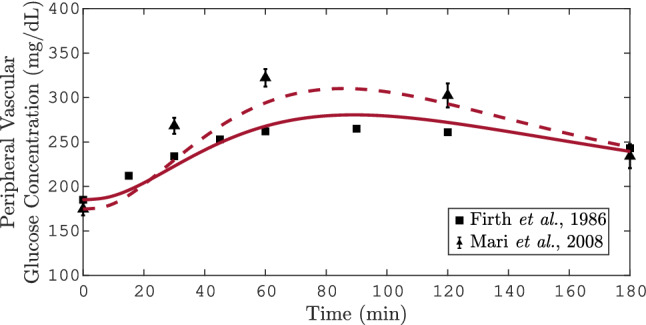
Simulation of 25 g and 75 g-OGTT. In this plot the solid and dashed lines represent the simulation of the blood glucose in the peripheral compartment of the T2DM model during a 25 g and 75 g-OGTT, respectively. The symbols represent the mean value of solute from the n subjects. Particularly, the triangles represent the mean ±SEM. The data for the 25 g and 75 g-OGTT were taken from Mari et al., and Firth et al., respectively.

Figure 4 shows the T2DM model response of the OGTT for different doses. Similarly to the observed clinical data, the model response $x_6$ rises to a maximum peak approximately at 80 min after the stimulus of 50 g and 75 g. The statistical value $\sigma$ for the 50 and 75 g-OGTT is 16.84 mg dL$^{-1}$, and 13 mg dL$^{-1}$, respectively. As can be seen, after oral glucose intake, the response of the model for the OGTT test is relatively slow, showing a maximum peak at approximately 80 min after glucose stimulation. Compared with the results of the PGIGI, the increase in glycaemia in the OGTT is slower. This is because of the digestion process, after an oral glucose intake, induces a delay proportional to the glucose appearance rate in the gut. Furthermore, as can be seen in Figs. 2, 3 and 4 the basal blood glucose is slightly elevated compared to the concentration of a healthy subject.

According to the World Health Organization guidance for diagnostic tests of DM, a fasting glucose concentration $\ge$126 mg dL$^{-1}$ is characteristic of DM^43^. Moreover, the patients with this impaired blood glucose should undergo by a formal 75 g-OGTT for DM diagnosis^43^. A representation of this test can be seen in Fig. 4 where after two-hour postload glucose the dotted curve had shown a glucose concentration $\ge$140 mg dL$^{-1}$. This is a characteristic behavior of DMT2 that contrasts with that of a healthy subject, where the normal homeostatic glucose process results in a concentration of less than 140 mg dL$^{-1}$ after 2 h of the glucose intake.

Based on the values of the $\sigma$ function, it can be concluded that the model emulates with acceptable precision what is reported in the clinical data for PGIGI, and OGTT. However, this model considers only the carbohydrate metabolism but not fat, and protein metabolism. Therefore, the effect of free fatty acids, and the physiology related to amino acids level on blood glucose dynamics are not included. Besides, the model does not consider the counter-regulatory effect of growth hormones, adrenaline, or cortisol. Nevertheless, the above can be considered later in the model by adding other subsystems for the free fatty acids dynamics, and other metabolic functions to consider the effect of the missing hormones.

## Concluding remarks

The main contribution of this article was derivating a model for T2DM, including physiological features to emulate blood glucose dynamics. The modelling departs from a PB-PKPD modelling approach, and the individual fitting of the sensitive metabolic rates allows us to capture the pathophysiology of the metabolic rates in T2DM. This methodological procedure enables us to successfully emulate the blood glucose dynamics of T2DM after a continuous intravenous glucose infusion an oral glucose intake. As convincing numerical evidence of the above, Figs. 3 and 4 show to what extent the T2DM model predicts the clinical data.

The individual fitting of the sensitive metabolic rates to clinical data ensures that the pathophysiology of T2DM is preserved, such that diverse scenarios might be predicted. For instance, this model can be used to determine appropriate oral therapy for blood glucose regulation by connecting a PKPD model of a hypoglycaemic drug (e.g., sulfonylureas, biguanides, thiazolidinediones, among others). Such is the case of metformin therapy, where the target metabolic rates were modified by adding a multiplicative factor in $r_{HGP}$, $r_{PGU}$, and $r_{GGU}$^44^. Similarly, the mathematical model we present lights up some complementarity to other research approaches. For example, the recent finding in improved glucose metabolism due to continued treatment with deuterium-depleted water (DDW) content in patients with T2DM^45^ could be emulated with an organ-based model. Again, it is feasible to consider a multiplicative factor to the metabolic rate $r_{PGU}$ to reproduce the alteration on peripheral glucose disposal, as indicated in some clinical researches^46^. Furthermore, this model can be used to develop a feedback model-based controllers for blood glucose regulation in T2DM patients. This idea triggers the possibility to achieve the normoglycaemia by means of single or combined therapy of oral hypoglycaemic agents with an exogenous insulin input connected to $r_{IVI}$. Finally, as a consequence of its mathematical structure, it is possible to consider structured or unstructured uncertainties in the described physiological-based model. Therefore, we can employ robust control techniques such as $H_\infty$ theory.

## Supplementary information

Supplementary Information.
