# Supplementary material for: Mathematical model of blood glucose dynamics by emulating the pathophysiology of glucose metabolism in type 2 diabetes mellitus
Source: Sci Rep. 2020 Jul 29;10:12697. doi: 10.1038/s41598-020-69629-0 (PMC7391357; doi:10.1038/s41598-020-69629-0)
Supplement: Supplementary file 1 — Supplementary Information. [file 41598_2020_69629_MOESM1_ESM.pdf]

Mathematical model of blood glucose dynamics by emulating the pathophysiology of glucose metabolism in type 2 diabetes mellitus

Nelida Elizabeth López-Palau[1,2] and José Manuel Olais-Govea[2,3]

[1]*División de Matemáticas Aplicadas, IPICyT, Camino a la Presa San José No. 2055, Lomas Cuarta Sección, 78216, San Luis Potosí, SLP, Mexico*

[2]*Tecnologico de Monterrey, Escuela de Ingeniería y Ciencias, Av. Eugenio Garza Sada 300, 78211, San Luis Potosí, SLP, Mexico.*

[3]*Tecnologico de Monterrey, Writing Lab, TecLab, Vicerrectoría de Investigación y Transferencia de Tecnología, Monterrey 64849, NL, Mexico.*

. .

I. SUBSYSTEMS OF THE MODEL

A graphical representation of each subsystem of the model, in Alverhag and Martin [1], is shown in Figs. S1-S3, where red arrows represent arterial blood, while blue arrows represent venous blood. Note that the blood cycle closes in the hearth and lungs compartment. Purple filled circles represent inputs or outputs (*i.e.*, measurements) of the system. The glucose and insulin subsystems, *i.e.*, in figs S1, and S2, respectively, are divided into several compartments representing organs or tissues interconnected trough blood flow, and whose direction is indicated by straight arrows. The subsystems of glucagon and incretins (Figs. S3(a), and S3(b), respectively) are divided into one compartment representing the whole body.

A general schematic representation of a compartment is shown in Fig. S4. In this compartment, C_A and C_B represent variables that quantify the concentration of solute in the sub-compartment of capillary blood, and the sub-compartment of interstitial fluid, respectively. The quantification of the solute in each compartment is carried out by means of a mass balance. The solute accumulation is a sum of exchange rates (*i.e.*, convection and diffusion), while its addition or elimination is due to metabolic rates. The following equations give the quantification of the solute for the sub-compartment of Fig. S4:

$$V_A\dot{C}_A = Q_I(C_i - C_A) + \frac{V_B}{T_{AB}}(C_B - C_A) \tag{S1}$$

$$V_B\dot{C}_B = \frac{V_B}{T_{AB}}(C_A - C_B) - r_{sink} + r_{source}, \tag{S2}$$

where C_i is the arterial concentration of solute, Q_I, is the volumetric blood flow rate, V_A and V_B are the blood volume of each sub-compartment, and T_B is the transcapillary diffusion time in the compartments of Figs. S1 and S2. The continuous line is used to indicate a low permeability between the walls, which divide the sub-compartments (*i.e.*, variable mass concentration), while the dashed line indicate a high permeability (*i.e.*, uniform mass concentration).

Specifically, the metabolic rates in the glucose, and glucagon subsystems are multiplicative functions with the following general form:

$$r = M^G M^I M^\Gamma r^B \tag{S3}$$

where r^B represents the basal value of the metabolic rate r, and each M is the isolated effect of the normalized concentration of glucose (M^G), insulin (M^I), and glucagon (M^Γ) of the normalized metabolic rate ($r^N = r/r^B$). To represent the characteristic sigmoidal non-linearities of biological data correlations, excepting the isolated effects that are states of the system (*i.e.*, M^I_{HGP} and M^I_{HGU}), all the isolated effects are hyperbolic tangent functions of some normalized component of the state, this is:

$$M(x_i^N) = \eta_{j_1} + \eta_{j_2}\tanh(\eta_{j_3}(x_i^N + \eta_{j_4})) \tag{S4}$$

where $x_i^N = x_i/x_i^B$ for $i \in \{1,2,...28\}$, and $\eta_{j_1}, \eta_{j_2}, ..., \eta_{j_4} \in H$ with $j_1, j_2...j_4 \in \mathbb{N} \leq 67$ are dimensionless parameters. In what follows, each of these subsystems, and its compartments and nomenclature is explained.

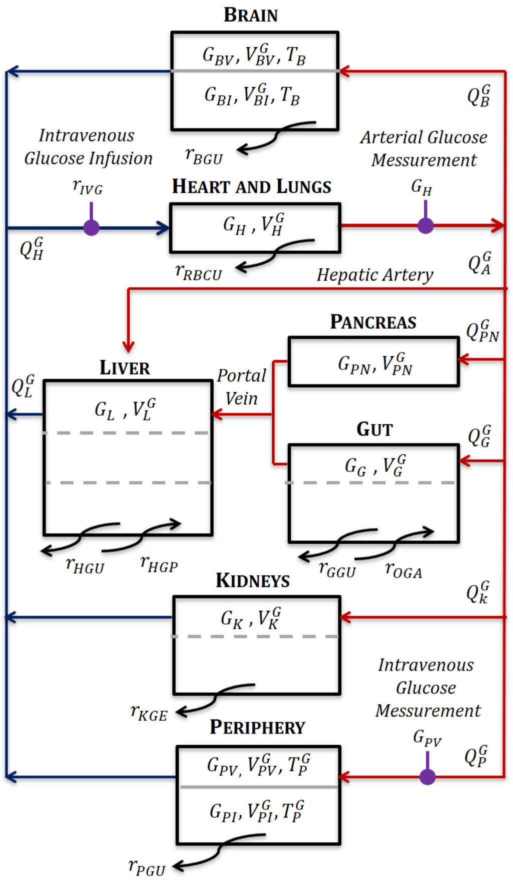

Figure S 1. Glucose subsystem diagram. This system is divided into several compartments representing organs or tissues interconnected trough blood flow, and whose direction is indicated by straight arrows. Red arrows represent arterial blood, while blue arrows represent venous blood. The purple dots represent inputs or outputs (*i.e.*, measurements) of the system. The metabolic rates that add or eliminate the mass of the subsystems are represented by curved arrows entering or leaving the compartments. The permeability between the walls of all compartments, excepting the brain and periphery, is relatively low. Therefore all the sub-compartments in these compartments can be considered as one. On the other hand, the compartments of the brain, and peripheral tissues present a high glucose permeability between the walls dividing sub-compartments; therefore, two well-defined spaces are considered.

A. Glucose Model

The mass balance in the sub-compartments of the glucose subsystem of the model has given rise to a set of 9 differential equations that are described in Table SI. The glucose subsystem considers 8 metabolic rates: 6 sinks and 2 sources of glucose. The metabolic rates of the brain glucose uptake, red blood cells glucose uptake, and gut glucose uptake are constants. This is $r_{BGU} = \eta_{21}$, $r_{RBCU} = \eta_{22}$, and $r_{GGU} = \eta_{23}$, respectively. The metabolic rates of peripheral glucose uptake (r_{PGU}), hepatic glucose production (r_{HGP}), and hepatic glucose uptake (r_{HGU}) are modeled as multiplicative effects of their basal rate as follows:

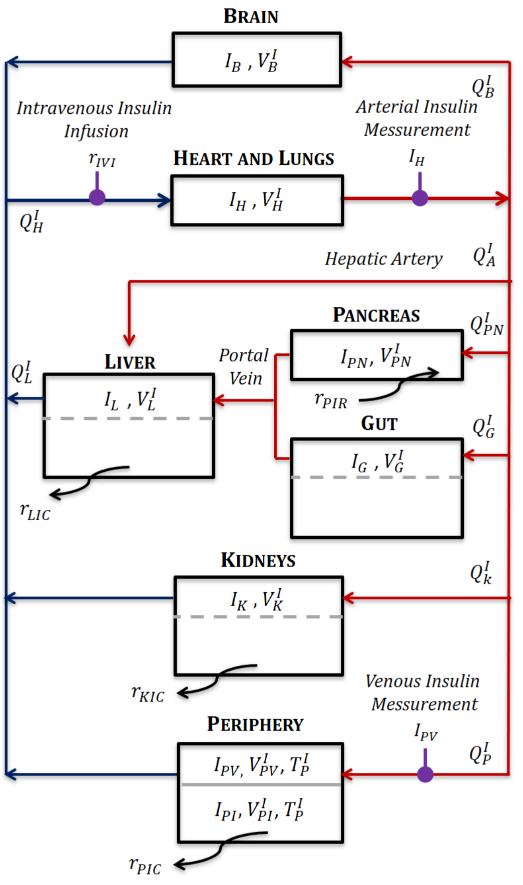

Figure S 2. Insulin subsystem diagram. Similar to Figure 1, excepting the periphery, the permeability between the walls of all compartments is relatively low, which implies that all the sub-compartments in these compartments can be considered as one. In this case, the compartment of the peripheral tissues considers a high glucose permeability between the walls dividing sub-compartments, and therefore, two well-defined spaces are considered.

1. The Equation (S3) defines the multiplicative function r_{PGU}, with $r^B_{PGU} = \eta_{15}$. The function r_{PGU} is mediated just by changes in the insulin, and glucose concentrations, then the effect of glucagon in Equation (S3) is considered as $M^{\Gamma}_{PGU} = 1$. The isolated effects of glucose, and insulin on the peripheral glucose uptake are determined by fitting the functions to experimental data [2]. Here, M^G_{PGU} is represented as a linear function passing through the origin (*i.e.*, $M^G_{PGU} = G_{PI}/G^B_{PI}$), and the Equation (S4) determines the function $M^I_{PGU}(I_{PI})$, with $\eta_j = \{\eta_{17}, \eta_{18}, \eta_{19}, -\eta_{20}\}$.

2. The multiplicative function r_{HGP} is defined by the Equation (S3) with $r^B_{HGP} = \eta_1$. As the insulin effect on the hepatic glucose production changes from basal to steady-state in an exponential way, this function is defined as:

$$\dot{M}^I_{HGP} = (M^{I_\infty}_{HGP} - M^I_{HGP})/\tau_I. \tag{S5}$$

The normalized rate of hepatic glucose production resulting from the glucagon step change is modeled by considering the function F_2 that serves to degrade the maximal response of glucagon action to half its initial impact. The above is $M^{\Gamma}_{HGP} = M^{\Gamma_0}_{HGP} - F_2$ where the function F_2 is defined from:

$$\dot{F}_2 = ((M^{\Gamma_0}_{HGP} - 1)/2 - F_2)/\tau_{\Gamma}. \tag{S6}$$

The isolated effect of glucose, the isolated steady-state effect of insulin, and the initial isolated effect of glucagon on the hepatic glucose production were determined by fitting the functions to clinical data [2], where $M^G_{HGP}(G_L)$, $M^{I_\infty}_{HGP}(I_L)$, and $M^{\Gamma_0}_{HGP}(\Gamma)$ are defined as in Equation (S4), with $\eta_j = \{\eta_4, -\eta_5, \eta_6, -\eta_7\}$, $\{0, \eta_2, \eta_3, 0\}$ and $\{\eta_{54}, -\eta_{55}, \eta_{56}, -\eta_{57}\}$, respectively.

3. The Equation (S3) defines the multiplicative function r_{HGU}, with $r^B_{HGU} = \eta_{11}$. The function r_{HGU} is mediated just by changes in the insulin and glucose concentrations, then the effect of glucagon in Equation (S3) is considered as $M^{\Gamma}_{PGU} = 1$. As the time course of mediated hepatic glucose uptake changes from its basal to its steady state value in an exponential manner with time,

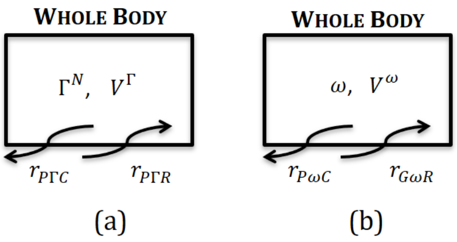

Figure S 3. Glucagon e incretins diagrams. Figures (a) and (b) shown the compartmental diagram of the glucagon, and incretins subsystem, respectively. In both subsystems, the entire body is considered as a single compartment.

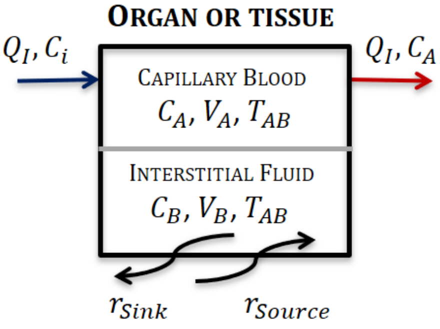

Figure S 4. The general outline of a compartment according to Alverhag and Martin model [1]. In this representation, there are two well-defined spaces: the capillary blood space, and the interstitial fluid space. Among them, there is a low permeability determined by the transcapillary diffusion time T_{AB}. The metabolic rates that add or eliminate the mass of the subsystems are represented by curved arrows entering or leaving the compartments, respectively.

Brain
$\dot{G}_{BV} = [Q_B^G(G_H - G_{BV}) - V_{BI}^G(G_{BV} - G_{BI})/T_B^G]/V_{BV}^G$
$\dot{G}_{BI} = [V_{BI}^G(G_{BV} - G_{BI})/T_B^G - r_{BGU}]/V_{BI}^G$
**Heart and Lungs**
$\dot{G}_H = [Q_B^G G_{BV} + Q_L^G G_L + Q_K^G G_K + Q_P^G G_{PV} - Q_H^G G_H - r_{RBCU} + r_{IVG}]/V_H^G$
**Liver**
$\dot{G}_L = [Q_A^G G_H + Q_G^G G_G + Q_{PN}^G G_{PN} - Q_L^G G_L + r_{HGP} - r_{HGU}]/V_L^G$
**Kidneys**
$\dot{G}_K = [Q_K^G(G_H - G_K) - r_{KGE}]/V_K^G$
**Periphery**
$\dot{G}_{PV} = [Q_P^G(G_H - G_{PV}) - V_{PI}(G_{PV} - G_{PI})/T_P^G]/V_{PV}^G$
$\dot{G}_{PI} = [V_{PI}(G_{PV} - G_{PI})/T_P^G - r_{PGU}]/V_{PI}$
**Gut**
$\dot{G}_G = [Q_G^G(G_H - G_G) - r_{GGU} + r_{OGA}]/V_G^G$
**Pancreas**
$\dot{G}_{PN} = Q_{PN}^G(G_H - G_{PN})]/V_{PN}^G$

Constants and variables	First Subscript
G =Glucose concentration (mg/dl)	B =Brain
Q =Vascular blood water flow rate (dl/min)	H =Heart and lungs
r =Metabolic source or sink rate (mg/min)	PN =Pancreas
T =Transcapillary diffusion time (min)	L =Liver
V =Volume (dl)	G =Gut
**Metabolic rates subscript**	K =Kidney
BGU =Brain glucose uptake	P =Periphery
GGU =Gut glucose utilization	A =Hepatic artery
HGP =Hepatic glucose production	
HGU =Hepatic glucose uptake	**Second Subscript**
KGE =Kidney glucose excretion	I =Interstitial Space
PGU =Peripheral glucose uptake	V =Vascular Space
$RBCU$ =Red blood cells glucose uptake	
IVI =Intravenous glucose infusion	**Superscript**
OGA =Oral glucose gastric absorption	G =Glucose model

Table S I. The upper part of the table shows the set of equations that are derived from the glucose subsystem. These equations are presented based on the compartment to which they correspond. The down part of the table shows the nomenclature used to define the states, and the parameters of the glucose subsystem.

Brain
$\dot{I}_B = Q_B^I(I_H - I_B)/V_B^I$
**Heart and Lungs**
$\dot{I}_H = [Q_B^I I_B + Q_L^I I_L + Q_K^I I_K + Q_P^I I_{PV} - Q_H^I I_H + r_{IVI}]/V_H^I$
**Liver**
$\dot{I}_L = [Q_A^I I_H + Q_G^I I_G - Q_L^I I_L + Q_{PN}^I I_{PN} - r_{LIC}]/V_L^I$
**Kidneys**
$\dot{I}_K = [Q_K^I(I_H - I_K) - r_{KIC}]/V_K^I$
**Periphery**
$\dot{I}_{PV} = [Q_P^I(I_H - I_{PV}) - V_{PI}^I(I_{PV} - I_{PI})/T_P^I]/V_{PV}^I$
$\dot{I}_{PI} = [V_{PI}^I(I_{PV} - I_{PI})/T_P^I - r_{PIC}]/V_{PI}^I$
**Gut**
$\dot{I}_G = Q_G^I(I_H - I_G)/V_G^I$
**Pancreas**
$\dot{I}_{PN} = [Q_{PN}^I(I_H - I_{PN}) + r_{PIR}]/V_{PN}^I$

Constants and variables	First Subscript
I =Insulin concentration (mU/l)	B =Brain
Q =Vascular plasma flow rate (l/min)	H =Heart and lungs
r =Metabolic source or sink rate (mU/min)	PN =Pancreas
T =Transcapillary diffusion time (min)	L =Liver
V =Volume (l)	G =Gut
**Metabolic rates subscript**	K =Kidney
KIC =Kidney insulin clearance	P =Periphery
LIC =Liver insulin clearance	A =Hepatic artery
PIC =Pancreas insulin clearance	**Second Subscript**
PIR =Pancreas insulin release	I =Interstitial Space
IVI =Intravenous insulin infusion	V =Vascular Space
	**Superscript**
	I =Insulin model

Table S II. The upper part of the table shows the set of equations that are derived from the insulin subsystem. These equations are presented based on the compartment to which they correspond. The down part of the table shows the nomenclature used to define the states, and the parameters of the insulin subsystem.

the function of the isolated effect of insulin is:

$$\dot{M}_{HGU}^{I} = (M_{HGU}^{I_\infty} - M_{HGU}^{I})/\tau_I \tag{S7}$$

The isolated effect of glucose, and the isolated steady-state effect of insulin on hepatic glucose uptake are determined by fitting the functions to clinical data [2], where $M_{HGU}^{G}(G_L)$, and $M_{HGU}^{I_\infty}(I_L)$ are defined by the Equation (S4), with $\eta_j = \{\eta_8, \eta_{60}, \eta_9, -\eta_{10}\}$ and $\{0, \eta_{52}, \eta_{53}, 0\}$, respectively.

Furthermore, the kidney glucose excretion is modeled as a hyperbolic tangent considering the rate of glucose filtration minus the rate of glucose reabsorption. This is defined as:

$$r_{KGE} = \left\{ \begin{array}{ll} \eta_{12} + \eta_{61} tanh(\eta_{13}(G_K - \eta_{14})) & 0 \leq G_K < 460 \\ \eta_{58} + \eta_{59} G_K & G_K \geq 460 \end{array} \right\} \tag{S8}$$

The rate of glucose absorption from the intestines into the blood, r_{OGA}, is obtained from the following equation:

$$\dot{r}_{OGA} = G_s/(T_A T_{GE}) - r_{OGA}/T_A, \tag{S9}$$

where the amount of glucose entering the stomach after a meal (OGC_S) depends on the content of carbohydrate of the meal (OGC_O). The above is represented by:

$$\begin{aligned} OGC_s = (OGC_0/\eta_{66})((t-\eta_{66})u(t-\eta_{66}) \\ -(t-\eta_{66}-1)u(t-\eta_{66}-1) \\ -(t-\eta_{66}-4)u(t-\eta_{66}-4)+(t-\eta_{66}-5)u(t-\eta_{66}-5)). \end{aligned} \tag{S10}$$

After glucose intake, the gastric emptying process rate describes the rate of glucose transference from the stomach to the intestines by:

$$\dot{G}_s = OGCS - G_s/T_{GE}. \tag{S11}$$

B. Insulin Subsystem

The graphical representation of the insulin subsystem of the model can be seen in Fig. S2. The mass balance in the sub-compartments has given rise to a set of 8 differential equations that are described in Table SII. In the insulin subsystem, 4 metabolic rates are considered: 3 sinks and 1 source. All the metabolic rates of insulin clearance are modeled as a fraction of the insulin quantity per minute that an

organ receives. This is: $r_{LIC} = F_{LIC}(Q_A^I I_H + Q_G^I I_G + Q_{PN}^I I_{PN})$, $r_{KIC} = F_{KIC} Q_K^I I_H$, and $r_{PIC} = I_{PI}/(((1-F_{PIC})/(F_{PIC}Q_P^I)) - T_P^I/V_{PI}^I)$. Whereas, the metabolic rate of pancreatic insulin release (r_{PIR}) is compartmentally modeled to reproduce the two characteristic phases of insulin secretion.

The model to emulate r_{PIR} comes from the fact that one large storage compartment in exchange for insulin with one small compartment. The quantity of insulin in the large compartment is considered constant. Conversely, the quantity of insulin in the small compartment (Q) is considered variable, sensitive to glucose concentration, and depending on either the rate of insulin exchange between compartments and the insulin secretion rate (S), this is:

$$\dot{Q} = \eta_{42}(\eta_{43} - Q) + \eta_{44}P - S, \tag{S12}$$

with P a function defining the rate of insulin provision from the large compartment to the small one. The above is obtained from:

$$\dot{P} = \eta_{35}(P_\infty - P), \tag{S13}$$

where P_∞ is the steady-state effect of glucose and incretins on insulin release. This is, $P_\infty = Y = X^{\eta_{40}} + \eta_{62}\omega$, where the function X represent an exciter of insulin secretion stimulated by glucose concentration. It is given by $X = G_H/(\eta_{37}^{\eta_{36}} + \eta_{38}G_H^{\eta_{39}})$. Subsequently, the insulin secretion rate is a function defined as:

$$S = (\eta_{46}(X-I)^{0^+} + \eta_{45}Y + \eta_{63}\omega)Q, \tag{S14}$$

where the notation $(X-I)^{0^+}$ means that the value of the function $X-I$ counts for S if it is positive but zero otherwise, and I represent an inhibitory entity (I) of insulin secretion. The last one is obtained from:

$$\dot{I} = \eta_{41}(X - I). \tag{S15}$$

From the Equation (S14) can be observed that the first term of the equation represents the first phase of insulin secretion as a proportion of the difference between X and I. Meanwhile, the second phase of insulin is obtained from the second term of the Equation (S14) as the slow transfer of insulin from the large compartment. The above means that it is controlled directly by the provision factor, and the amount of insulin from the labile. Subsequently, the third term of the Equation (S14) reflects the effect of the concentration of the incretin over insulin release.

The metabolic rate of insulin release is modeled as a scale of the basal insulin secretion rate (r_{PIR}^B) as follows:

$$r_{PIR} = S(G_H)/S(G_H^B)r_{PIR}^B, \tag{S16}$$

where r_{PIR}^B is determined by the fasting insulin concentration in the body as $r_{PIR}^B = I_H^B(Q_H^I/(1-F_{LIC}) - Q_A^I - Q_G^I - (1-F_{PIC})/(1-F_{LIC})Q_P^I - Q_B^I/(1-F_{LIC}) - Q_K^I(1-F_{KIC})/(1-F_{LIC}) - Q_{PN}^I)$.

Whole Body Glugacon	
	$\dot{\Gamma} = \eta_{33}[r_{P\Gamma R}^N - \Gamma]/V^\Gamma$
**Constants and variables**	**Superscript**
Γ =Glucagon concentration (pg/ml)	Γ =Glucagon model
V =Volume (ml)	N =Normalized value
r =Metabolic source or sink rate (pg/min)	
**Metabolic rates subscript**	
$P\Gamma R$ = Pancreatic glucagon release	
**Whole Body Incretins**	
	$\dot{\omega} = [r_{G\omega R} - r_{P\omega C}]/V^\omega$
	$\dot{\omega}_G = \eta_{65}OGC_s - r_{G\omega R}$
**Constants and variables**	
	ω =Incretins concentration above normal levels (pmol/l)
	ω_G =Quantity of incretins in the gut above normal (pmol/l)
	r =Metabolic source or sink rate (pmol/min)
	V =Volume (l)
**Metabolic rates subscript**	**Superscript**
$G\omega R$ = Gut incretins release	ω =Incretins model
$P\omega C$ =Plasma incretins clearance	N =Normalized value

Table S III. The upper and down part of the table shows the set of equations that are derived from the glucagon and incretins subsystem, and the nomenclature used to define the states and the parameters of the respective subsystem.

C. Glucagon and incretins subsystem

The graphical representation of the glucagon and incretins subsystems can be seen in Figs. S3(a) and S3(b), respectively. The mass balance in the sub-compartments has given rise to a set of 3 differential equations described in the Table SIII. Both subsystems have a metabolic sink, representing the clearance in blood plasma, and a metabolic source, representing release into blood plasma. The rates of glucagon and incretins clearance and the rate of incretins release are modeled as the following linear functions: $r_{P\Gamma C} = \eta_{33}\Gamma$, $r_{P\omega C} = r_{M\omega C}\omega$, and $r_{G\omega R} = \omega_G/T_\omega$, respectively. Conversely, the normalized rate of glucagon release ($r_{P\Gamma R}^N$) is modeled as the multiplicative function defined by $r_{P\Gamma R}^N = M_{P\Gamma R}^G M_{P\Gamma R}^I$. In the above, the isolated effect of glucose ($M_{P\Gamma R}^G(G_H^N)$), and insulin ($M_{P\Gamma R}^I(I_H^N)$) on glucagon release are determined in [2] by fitting the Equation (S4) to experimental data with $\eta_j = \{\eta_{24}, -\eta_{25}, \eta_{26}, -\eta_{27}\}$ and $\eta_j = \{\eta_{28}, -\eta_{29}, \eta_{30}, -\eta_{31}\}$, respectively.

II. PARAMETERS OF THE MODEL

The upper and down part of Table SIV contains all the parameters and its nominal value for the hemodynamical, and metabolic processes of the model, respectively [1]. For model simulation, when the value of the nominal parameter is considered, then the blood glucose dynamics of a healthy human body is reproduced.

Parameters of the hemodynamical processes (π)			
$V^G_{BV} = 3.5$	$V^I_K = 0.505$	$Q^G_G = 9.6$	$F_{KIC} = 0.30$
$V^G_{BI} = 4.5$	$V^I_{PV} = 0.735$	$Q^G_{PN} = 0.5$	$F_{PIC} = 0.15$
$V^G_H = 13.8$	$V^I_{PI} = 6.3$	$Q^G_A = 2.5$	$T^G_B = 2.1$
$V^G_L = 23.5$	$V^I_G = 0.945$	$Q^I_B = 0.45$	$T^G_P = 5$
$V^G_K = 6.6$	$V^I_{PN} = 0.07$	$Q^I_H = 3.12$	$T_A = 22$
$V^G_{PI} = 63$	$V^\Gamma = 9930$	$Q^I_L = 0.9$	$T^I_P = 20$
$V^G_{PV} = 10.4$	$V^\omega = 9.930$	$Q^I_K = 0.72$	$T_\omega = 25$
$V^G_G = 11.2$	$Q^G_B = 5.9$	$Q^I_P = 1.05$	$T_{GE} = 73$
$V^G_{PN} = 1.6$	$Q^G_H = 43.7$	$Q^I_G = 0.684$	$\tau_I = 25$
$V^I_B = 0.265$	$Q^G_L = 12.6$	$Q^I_{PN} = 0.036$	$\tau_\Gamma = 65$
$V^I_H = 0.985$	$Q^G_K = 10.1$	$Q^I_A = 0.18$	
$V^I_L = 1.07$	$Q^G_P = 15.1$	$F_{LIC} = 0.40$	
Parameters of the metabolic rates (η)			
$\eta_1 = 155$	$\eta_{18} = 6.52$	$\eta_{35} = 0.0482$	$\eta_{52} = 2$
$\eta_2 = 2.7$	$\eta_{19} = 0.338$	$\eta_{36} = 3.27$	$\eta_{53} = 0.55$
$\eta_3 = 0.39$	$\eta_{20} = 5.82$	$\eta_{37} = 132$	$\eta_{54} = 1.21$
$\eta_4 = 1.42$	$\eta_{21} = 70$	$\eta_{38} = 5.93$	$\eta_{55} = 1.14$
$\eta_5 = 1.41$	$\eta_{22} = 10$	$\eta_{39} = 3.02$	$\eta_{56} = 1.66$
$\eta_6 = 0.62$	$\eta_{23} = 20$	$\eta_{40} = 1.11$	$\eta_{57} = 0.89$
$\eta_7 = 0.497$	$\eta_{24} = 2.93$	$\eta_{41} = 0.931$	$\eta_{58} = 330$
$\eta_8 = 5.66$	$\eta_{25} = 2.10$	$\eta_{42} = 0.00794$	$\eta_{59} = 0.872$
$\eta_9 = 2.4$	$\eta_{26} = 4.18$	$\eta_{43} = 6.33$	$\eta_{60} = 5.66$
$\eta_{10} = 1.48$	$\eta_{27} = 0.61$	$\eta_{44} = 0.575$	$\eta_{61} = 71$
$\eta_{11} = 20$	$\eta_{28} = 1.31$	$\eta_{45} = 0.00797$	$\eta_{62} = 0.003$
$\eta_{12} = 71$	$\eta_{29} = 0.61$	$\eta_{46} = 0.136$	$\eta_{63} = 0.0001$
$\eta_{13} = 0.011$	$\eta_{30} = 1.06$	$\eta_{47} = G^B_H$	$\eta_{64} = 0.14$
$\eta_{14} = 460$	$\eta_{31} = 0.47$	$\eta_{48} = G^B_L$	$\eta_{65} = 0.009$
$\eta_{15} = 35$	$\eta_{32} = 9.11$	$\eta_{49} = I^B_{PI}$	$\eta_{66} = 4$
$\eta_{16} = G^B_{PI}$	$\eta_{33} = 910$	$\eta_{50} = I^B_H$	$\eta_{67} = 0$
$\eta_{17} = 7.03$	$\eta_{34} = 18.69$	$\eta_{51} = I^B_L$	

Table S IV. Nominal values of the parameters in the mathematical model proposed in [1].

[1] Alverhag, K., & Martin, C. The feedback control of glucose: on the road to Type II diabetes, *Proceedings of the 45 IEEE Conference on Decision and Control*, San Diego, pp. 685-690, https://doi.org/10.1109/CDC.2006.377192, (2006).

[2] Sorensen. J. T. A Physiological Model of Glucose Metabolism in Man and its use to Design and Assess Improved Insulin Therapies for Diabetes. *Ph.D. Thesis, Massachusetts Institute of Technology*, (1985).
